# Revisiting the ‘direct mineral cycling’ hypothesis: arbuscular mycorrhizal fungi colonize leaf litter, but why?

**DOI:** 10.1038/s41396-019-0403-2

**Published:** 2019-03-25

**Authors:** Rebecca A. Bunn, Dylan T. Simpson, Lorinda S. Bullington, Ylva Lekberg, David P. Janos

**Affiliations:** 1 Department of Environmental Sciences, Western Washington University, 516 High St., MS-9181, Bellingham, WA 98225 USA; 2 MPG Ranch, Missoula, MT USA; 3 Department of Ecosystem and Conservation Sciences, University of Montana, Missoula, MT USA; 4 Department of Biology, University of Miami, Miami, FL USA

**Keywords:** Symbiosis, Forest ecology, Microbial ecology, Fungal ecology, Plant ecology

## A hypothesis of ‘direct mineral cycling’

Half a century ago in an Amazonian rainforest, Went and Stark [1, 2] observed ‘endotrophic mycorrhizal’ hyphae proliferating in leaf litter adjacent to mycorrhizal roots. They inferred the fungi were accessing, and subsequently transferring, litter-bound mineral nutrients to host plants in a phenomenon they called ‘direct mineral cycling.’ This hypothesis was consistent with acceptance of fungi as primary decomposers in forest ecosystems and mycorrhizal fungi as symbionts that transfer mineral nutrients (hereafter, ‘nutrients’) to host plants. ‘Endotrophic mycorrhizas,’ as used by those authors, were later termed ‘arbuscular mycorrhizas’ and involve a phylogenetically distinct group of fungi from the phylum Glomeromycota [3]. Early observations of arbuscular mycorrhizal (AM) fungi proliferating in organic matter led researchers to surmise saprotrophic capabilities [4–6]. However, attempts to culture AM fungi independent of host plants were unsuccessful, and researchers now understand AM fungi as obligate symbionts entirely dependent on host plant roots for carbon [7, 8]. In fact, AM fungi appear incapable of producing the lytic enzymes necessary to cleave organic molecules [9, 10] and evidence still largely, but not universally, suggests these fungi are limited to assimilating inorganic forms of nutrients (nitrogen reviewed by Hodge and Storer [11]; phosphorus by Smith et al. [12]). Thus, AM fungi alone are unlikely to access litter-bound nutrients as Went and Stark [1, 2] originally envisioned, and ‘direct mineral cycling’ has languished in the bin of unsupported hypotheses.

Yet AM fungi colonize litter in a variety of habitats (Table 1), and AM host plants are often successful in organic soils despite predictions that these plants are largely restricted to mineral soils where inorganic nutrients predominate [13, 14]. In this Perspective, we argue for a reconsideration of the ‘direct mineral cycling’ hypothesis; not because we believe that these fungi can directly mobilize organically bound nutrients, but because of ample evidence that AM fungi can influence degradation of organic matter and subsequently acquire and transfer a portion of released nutrients to their associated host plants [15, 16]. Therefore, the functional consequences of AM fungi growing into leaf litter may match those of ‘direct mineral cycling.’ We review the literature and present new data that unequivocally show rapid colonization by AM fungi inside dead leaves in coniferous-dominated forests in the Pacific Northwest. Thus, we argue that AM fungal colonization of litter and not-yet-decomposed plant matter may be a global phenomenon that could have far-reaching implications for plant–plant interactions and nutrient cycling in both natural and managed ecosystems. Finally, we outline a set of questions that we hope will spur cross-disciplinary research into what we believe is an often overlooked and under-researched topic.

**Table 1:** Evidence of AM fungi in leaf litter has come from a variety of ecosystems but only seven publications. Data in this table are taken directly from referenced publications unless noted otherwise. Abbreviations are used for elevation (elev.), temperature (temp.), and months (mo.)

Type of evidence	Climate, habitat type	Location	Elev. (m)	Annual rainfall (mm)	Annual temp. (°C)	Type of study	Reference	Notes
Microscopy/morphological identification	Tropical, montane—deforested	Zipacón, Cundinamarca, Colombia 04°56’23” N; 73°50’11” W	2650	735	15	Natural litter	[28]	
	Tropical, montane—ranch	Guatavita, Cundinamarca, Colombia 04°46'06” N; 74°22’45” W	2550	1274	14	Natural litter	[28]	
	Tropical, montane—páramos	Subachoque, Cundinamarca, Colombia 04°55’54” N; 74°10’35” W	3450	865	13	Natural litter	[28]	a
	Tropical, riparian wet forest	Medina, Cundinamarca, Colombia 04°30’ N; 73°29’ W	240	3170	27	Natural litter	[66]	
	Tropical, evergreen lower montane forest	Cordillera Real, Ecuador 03°59’ S; 79°05’ W	2030–2120	2600	15	Natural litter	[67]	
	Subtropical, hardwood hammock	Miami, Florida, USA 25°39’57" N; 80°16’49" W	17	1400–1525	18–27	Litterbags 2.3–18 mo.	[68]	b
	Temperate, mixed maritime forest	Bellingham, Washington, USA 48°44’10” N; 122°28’53” W	150–210	910	10	Litterbags 2.5–5 mo.	This study	
	Temperate, mixed maritime forest	Bellingham, Washington, USA 48°39’17” N; 122°27’43” W	400–560	910	10	Litterbags 2.5–5 mo.	This study	
Molecular: ITS	Temperate, oak forest	Xaverovský Háj Natural Reserve, Prague, Czech Republic 50°05’38” N; 14°36’48” E	227	521	9	Litterbags 0–24 mo.	[26]	c
	Temperate, mixed temperate forest	Masaryk Forest Křtiny, Brno, Czech Republic 49°15’ N; 16°15’ W	488	400–500	8.5–9.0	Natural litter	[27]	d
Molecular: SSU	Temperate, mixed maritime forest	Bellingham, Washington, USA 48°44’10” N; 122°28’53” W	150–210	910	10	Litterbags 2.5–5 mo.	This study	
	Temperate, mixed maritime forest	Bellingham, Washington, USA 48°39’17” N; 122°27’43” W	400–560	910	10	Litterbags 2.5–5 mo.	This study	

^a^Mean annual temperature and rainfall taken from climate-data.org for Subachoque (https://en.climate-data.org/location/34084/; Accessed 10 Oct 2018)

^b^Coordinates and elevation provided by David Janos

^c^Elevation estimated by Google Earth (https://www.google.com/earth/; Accessed 10 Oct 2018) given the latitude and longitude coordinates

^d^Elevation estimated by Google Earth (https://www.google.com/earth/; Accessed 10 Oct 2018) given the latitude and longitude coordinates. Mean annual rainfall estimated from the 1961 to 1990 normals for Prague (https://usclimatedata.com/climate/czech-republic/ez#; Accessed 10 Oct 2018)

### Mycorrhizal fungi types in organic versus mineral soils: AM fungi defy the rules

Initially, the ‘direct mineral cycling’ hypothesis was appealing because it explained an incongruity in tropical forests. Tropical forest soils are notoriously infertile due to leaching and phosphorus-sorption, yet they support abundant plant biomass. A closed nutrient cycle could explain how plant biomass is maintained despite low levels of plant-available nutrients in soil. Although arbuscular mycorrhizas are known to improve host plant mineral nutrition in infertile soils, most nutrients in tropical systems are recycled from litter, and therefore, presumed to be initially inaccessible to AM fungi. Despite advances in mycorrhizal ecology, the success of AM hosts in tropical forests has remained a paradox.

In contrast to AM fungi, some ectomycorrhizal (EM) fungi seem capable of direct decomposition. EM fungi can produce lytic enzymes to release organically bound nutrients [17] and a growing body of evidence supports decomposer capabilities in some taxa [18]. This contrast suggests that EM, but not AM, fungi access organically bound nutrients. Read [13], and later Read and Perez-Moreno [14], proposed that this functional difference between mycorrhizal types explains the global distribution of their host plants. Under this framework, the abundance of AM fungi would be inversely related to concentrations of soil organic matter (SOM) and soil fertility would delineate the occurrence of AM plant species. If true, AM hosts should predominate at mid to low latitudes and low elevations where rapid decomposition results in thin litter layers and low SOM concentrations. Yet, AM hosts are prevalent throughout northern and central latitudes in Europe [19], and a global survey failed to find any correlation between SOM and abundance of AM fungi [20]. Further, AM fungi and their plant hosts can be widespread in SOM-rich ecosystems, including alpine meadows [21], tropical cloud forests [22], and the Arctic [23]. Although AM fungal community composition may shift with SOM at a global level [24] if not a landscape level [25], AM fungi and their hosts clearly are not restricted to mineral soils.

### AM fungi colonize leaf litter and recently fallen leaves across ecological regions

Proliferation of AM fungi in leaf litter (Table 1), well above the mineral soil horizon, further contradicts the predictions of Read [13] and Read and Perez-Moreno [14]. Evidence of this phenomenon has recently become even more compelling with molecular data indicating the presence of Glomeromycotan fungi in leaf litter in temperate forests (this study; [26, 27]). Not only do AM fungi occur in the litter horizon, they also may colonize plant leaves that have not yet substantially decomposed [28].

**Fig. 1:**
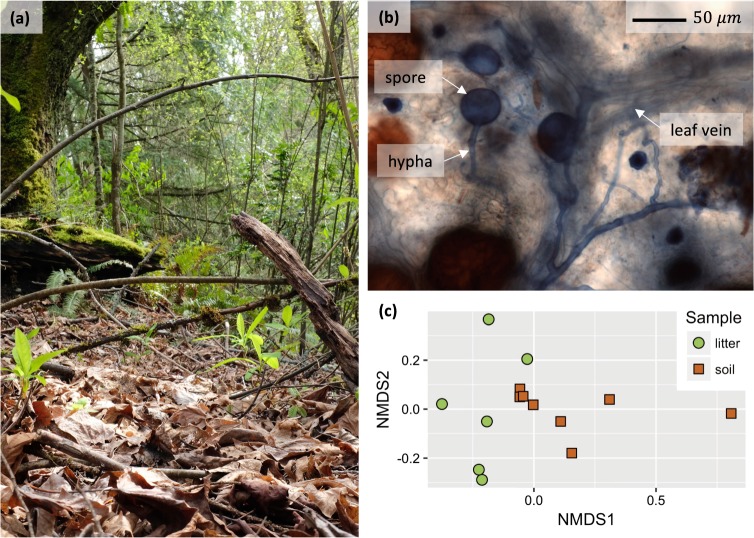
**a** Bigleaf maple (*Acer macrophyllum*) litter (foreground) on the floor of a temperate, North American, Pacific Northwest forest. Indian plum (*Oemleria cerasiformis*) and sword fern (*Polystichum munitum*) grow up through the litter. These leaves may have fallen from the mature bigleaf maple tree (seen here as a moss-covered, large trunk on the left), but bigleaf maple litter is also found in stands with the dominant overstory tree, ectomycorrhizal Douglas fir (*Pseudotsuga menziesii*; dark horizontal branches of Douglas fir are shown just to the right of the bigleaf maple trunk). **b** Arbuscular mycorrhizal (AM) fungus-like spore with associated hyphae as well as other fungus hyphae in bigleaf maple litter. Leaf veins also are visible. Litter was cleared and stained with Trypan blue and then viewed with light microscopy at 400 × . **c** Nonmetric multidimensional ordination of predominant arbuscular mycorrhizal fungi amplified from leaf litter and adjacent soil samples. Fungal communities amplified from litter and soil were distinct from each other (*p* = 0.004, Table S4). SSU sequence data were resampled to 114 sequences/sample. Samples with fewer sequences were dropped from this analysis. Full dataset (pre-rarefaction) is available in Table S2

In this Perspective, we add to previous work and unequivocally show rapid AM fungal colonization of dead leaves in mixed maritime forests dominated by the EM host, Douglas fir (*Pseudotsuga menziesii*; Fig. 1a, Supporting Information Table S1). We combined the inherent strengths of molecular (targeting the small subunit ribosomal RNA [rRNA] gene) and microscopic techniques. Microscopic observations can reveal spatial relationships and a measure of abundance, and molecular data can provide indisputable evidence that the observed fungi truly are Glomeromycotan. Using litterbags with newly fallen leaves (Fig S1) and cleaning leaf surfaces prior to molecular assessments of AM fungi using the AM fungi-specific primer pair WANDA-AML2 (Table S2), we observed AM fungus-like hyphae growing inside intact leaf litter within 3 months of litter bag deployment (Fig. 1b). Hyphal densities (based on morphological features) in leaf litter correlated with total sequence abundances of AM fungi in soil (*r* = 0.81, *t*_6_ = 3.4, *p* = 0.015; Fig S2). Concordantly, total sequence abundances of AM fungi in leaf litter correlated with those in soil (ln[*x* + 1] transformed; *r* = 0.76, *t*_6_ = 2.8, *p* = 0.030; Fig S3). This is logical given our understanding that the obligately root-associated fungi would extend between roots in the soil and leaf litter, and implies that AM fungi enter leaf litter near AM host plants. In addition, we found substantial variation in the number of AM fungal sequences in leaf litter among sites, which may reflect a patchy distribution of AM host plants in these forests. Dominant AM fungi virtual taxa (VT) assemblages in soil were distinct from those in leaf litter (Fig. 1c, Tables S3 and S4 Fig S4). However, VT detected only in leaf litter did not cluster phylogenetically, but instead were closely related and sometimes identical  to VT detected only in soil (Fig. S5). This suggests niche differentiation similar to what has been found with EM fungi [29] and fungi in general [27], but differentiation may occur within, rather than among, phylogenetic clusters. Importantly, our results show that AM fungi may enter leaf litter even in forests dominated by EM host plants.

Our data corroborate and strengthen previous findings that AM fungi colonize leaf litter across a wide array of ecological and climatic regions. Furthermore, AM fungi appear to penetrate new litter within a few months and AM fungal taxa may differ in their preference for leaves or soil. Our methods and full results can be found in Supplemental Information.

### What stimulates AM fungi to colonize leaf litter?

Perhaps we should not be surprised if AM fungi frequently colonize leaf litter. Although AM fungal hyphae typically grow through the soil matrix and in the cortical tissue of plant roots, they also have been observed within other substrates. AM fungal hyphae and vesicles have been found in plant parts other than root cortical tissues, i.e., root xylems and rhizome scales [30, 31], and AM fungal spores have been found within oribatid mites, dead seeds, and even other spores [32–35]. The propensity of AM fungi to grow into ‘tiny holes’ has long been inferred from their successful exploitation of such nutrient-rich soil microsites, and that attribute alone might explain the presence of AM fungi in these unusual substrates. However, these substrates all are ‘high quality’ organic materials (i.e., have low carbon to nitrogen ratios). Conventional views on AM fungi would predict fungal growth occurring after decomposition of the substrate has begun and at least some mineralization has occurred. However, our observation of active colonization of new leaf litter implies AM fungi instead might ‘recognize’ substrates as a source of nutrients prior to decomposition.

AM fungi responding to litter-bound nutrients may have been considered unlikely at one time, but this is changing. Although AM fungi are best known for scavenging inorganic phosphorus from the soil, they also can access organically bound phosphorus when grown with other soil biota [36], particularly in phosphorus-limited environments [37]. Furthermore, when nitrogen is limiting, AM fungi can sense and upregulate genes in response to organic nitrogen [38]. In fact, attraction to nitrogen may underlie the contrast between the typical positive growth response of AM fungi to high quality organic matter amendments (e.g., organic soil particles [8]; cattle manure [39]; and broadleaf litter [40, 41]) versus a neutral to negative response to low-quality amendments (e.g., cellulose [42–44]; but see [45, 46]). In some instances, AM fungi may find organically bound nutrients in litter even more accessible than inorganic forms in the underlying soil [47]. Thus, colonization of litter by AM fungi may occur when litter is rich in nutrients that are otherwise scarce in the soil matrix, even if those nutrients are organically bound.

### How can AM fungi access nutrients in leaf litter?

In the absence of other soil biota, AM fungi are unable to mobilize or access organically bound nutrients (i.e., under gnotobiotic conditions [48]; but see [49]). However, in the presence of other soil biota, AM fungi can accelerate decomposition of complex organic materials like grass litter [15, 50]. Thus, the effects of AM fungi on decomposition must be mediated through other organisms. In fact, experimental systems that have demonstrated transport of nutrients from organic matter to host plants via AM fungi typically include the methodological step of equalizing initial microbial communities across the mycorrhizal and non-mycorrhizal treatments (e.g., [15, 16, 51]). Consequently, this nutrient pathway most likely results from an interaction between AM fungi and the microbial community, rather than the fungi alone. In such interactions, AM fungi may release labile carbon into their hyphosphere, stimulating activity of microbial decomposers [52] and increasing degradation rates of SOM [53, 54] in a version of priming effects [55]. The involved microbes might be specialized, living primarily on hypha surfaces and forming a kind of secondary symbiosis with mycorrhizal fungi in which access to organically bound nutrients is traded for mycorrhizal carbon (i.e., ‘hypersymbionts,’ [56]). Alternatively, the microbes could be free-living organisms, which respond to any labile carbon source. Researchers are just beginning to untangle pathways from organically bound nutrients to AM fungi and to identify the involved organisms. For instance, while investigating pathways of nitrogen, Bukovská et al. [44] found a correlation between hyphal proliferation and prokaryotic ammonium oxidizer abundance, suggesting ammonium-oxidizers may control the availability of nitrogen to AM fungi. Yet, by including a non-mycorrhizal control in a subsequent study, Bukovská et al. [57] observed that ammonium oxidizers were actually repressed by AM fungi, and instead, eukaryotic protists appeared to be the important players in nitrogen recycling. Pathways for phosphorus, which include mutually beneficial interactions between AM fungi and phosphate-solubilizing bacteria [58], may be less complicated. In phosphorus-limited environments, AM fungi and free-living phosphate-solubilizing bacteria benefit each other by providing the essential carbon or phosphorus that the other needs [59], which can increase mineralization of organically bound phosphorus [60].

No matter the pathway, an active role of AM fungi in stimulating decomposition implies a tight coupling between AM fungi and decomposers, groups that have historically been considered separate.

### Consequences of AM fungi colonizing leaf litter and future research directions

Based on these cumulative research findings, we propose that Went and Stark’s [1, 2] hypothesis may be accurate regarding the net effects of AM fungal colonization of leaf litter despite a misunderstanding of the underlying mechanisms. If AM fungi really are cued to colonize leaf litter because the litter is a nutrient source, and subsequently promote decomposition through stimulation of other organisms, then colonization of leaf litter may be an important pathway for cycling nutrients from litter to host plant. This is the essence of the ‘direct mineral cycling’ hypothesis. If true, then this nutrient pathway bypasses the soil matrix and allows AM hosts to be successful in infertile soils, potentially explaining the tropical forests paradox. Furthermore, if AM hosts have access to organically bound nutrients, they may be more competitive with EM hosts in organic soils than previously recognized. Yet, the extent to which AM hosts have access to nitrogen acquired by AM fungi varies widely for reasons that are not yet understood [11]. Thus, the significance of this pathway requires further investigation. Nevertheless, by assimilating nutrients before they reach the soil matrix AM fungi likely increase overall nutrient retention within ecosystems. Indeed, AM fungi are known to dramatically decrease leaching losses of inorganic phosphorus [61] and nitrogen [62, 63]. Recent evidence suggests the same may also be true for organically bound nutrients [64], which may partially occur through AM fungal colonization of leaf litter.

Many aspects of these ideas are preliminary. We do not yet have comprehensive data about the geographic extent of the phenomenon of AM fungi in leaf litter or the abundance of AM fungi relative to fungal decomposers in leaf litter, nor do we understand the biotic and abiotic factors that affect such colonization. We do not know if AM fungi are frequently coupled with decomposers, or if the studies cited here are isolated cases. Quantifying the abundance of AM fungi in leaf litter using either quantitative PCR or neutral lipid fatty-acid analyses through time might begin to address such questions. We have yet to determine if nutrients immediately acquired from litter and transferred to host plants are of sufficient quantity to affect plant fitness. Moreover, many of the studies cited here (e.g., [15, 41, 43, 57, 59]) are based on only one or two AM fungal taxa. This is a serious handicap because the functional differences among AM fungi are not well understood [65] and community effects are difficult to predict from single taxon studies. Yet, all these limitations are open doors for future research. We present questions that we consider most pressing (Table 2), but additional lines of inquiry could be formulated. We believe full answers to these questions will be possible only with cooperation across a wide range of scientific skill sets. For instance, on a molecular level, biochemists and microbiologists are needed to identify the pathways by which nutrients may move from bound in leaf litter to being assimilated into the mycorrhizal symbiosis; biogeochemists are needed to quantify the relative magnitude of nutrients potentially cycled through this pathway; and on an organismal to landscape level, plant and soil ecologists are needed to determine if this nutrient pathway is significant for individual plants and if it helps explain the competitive success of AM hosts in forest ecosystems.

**Table 2:** Questions that need to be investigated to further understand the nutrient cycling in ecosystems, as well as plant distributions in specific ecosystems

Item no.	Questions
1	Is colonization of litter by arbuscular mycorrhizal (AM) fungi a global phenomenon that occurs across ecosystems that contain AM host plants?
2	In colonizing litter, do AM fungi minimize leaching losses of mineral nutrients in ecosystems with relatively rapid decomposition, such as lowland tropical forests on Oxisols and Ultisols?
3	Do AM fungi accelerate decomposition rates through multiple pathways including stimulation of microbial decomposers?
4	Do AM fungal taxa differ in their propensity to colonize organic matter? If so, are those differences related to their ability to recognize organic sources of nutrients and/or to promote decomposition?
5	Given evidence of AM fungi accelerating decomposition rates and transporting mineral nutrients from organic substrates to host plants, could we expect AM host plants to be competitive across both organic and mineral soils, including oligotrophic ecosystems with slow decomposition and accumulation of organic matter, and mixed-mycorrhiza forests, such as those of the Pacific Northwestern United States?

## Conclusions

We corroborate and extend findings of AM fungi colonizing leaf litter across climatic regions. In addition, we find different AM fungal taxa predominated within leaf litter and soil, suggesting that those fungi may differ in their tendency to colonize leaf litter, which could result in niche differentiation between substrates. Taking our findings together with previous work on AM fungi and organic substrates, we suggest that mycorrhiza research may be missing a large and important part of AM fungal function. Although root colonization by AM fungi and extraradical hyphal density in mineral soil are frequently quantified, all too rarely has the spread of AM fungal hyphae within litter been investigated. Such spread may be a ‘hidden’ indication of the importance of AM fungi in forest ecosystems where their functions may include promoting the release of nutrients from leaf litter and transferring nutrients to host plants, thereby retaining nutrient capital. If true, AM fungi are providing host plants access to nutrient pools hitherto thought to be limited to hosts of other types of mycorrhizal fungi, which may help explain the success of AM host plants in both organic and tropical soils.

## Supplementary information

Supplemental Methods and Results

Tables S1, S3, and S4

Table S2
