# Supplementary material for: Revisiting the ‘direct mineral cycling’ hypothesis: arbuscular mycorrhizal fungi colonize leaf litter, but why?
Source: ISME J. 2019 Mar 25;13(8):1891–8. doi: 10.1038/s41396-019-0403-2 (PMC6775977; doi:10.1038/s41396-019-0403-2)
Supplement: Supplementary file 1 — Supplemental Methods and Results [file 41396_2019_403_MOESM1_ESM.pdf]

Revisiting the 'direct mineral cycling' hypothesis: Arbuscular mycorrhizal fungi colonize leaf litter, but why?

Rebecca A. Bunn, Dylan T. Simpson, Lorinda S. Bullington, Ylva Lekberg, David P. Janos

The following Supporting Information is available for this article:

Supplemental Summary and Results. Summary of study providing new evidence of AM fungi colonizing leaf litter

Supplemental Methods. Detailed description of the field studies, PCR amplification and associated bioinformatics analyses, and statistical analyses.

Fig. S1. Leaf litter bags
Fig. S2. AM fungal sequence abundance in soil samples versus hyphal densities in overlying bigleaf maple leaf litter samples
Fig. S3. AM fungal sequence abundance in the soil versus palm leaf litter samples
Fig. S4. Species accumulation curves
Fig. S5. Neighbor-joining tree showing phylogenetic differences among recovered sequences of arbuscular mycorrhizal fungi.

Table S1. Characteristics of studied forests.
Table S2. Total (non-resampled) taxa sequences of recovered arbuscular mycorrhizal fungi.
Table S3. Arbuscular mycorrhizal fungal taxa found in leaves, soil, or both leaves and soil after resampling to 114 sequences/sample.
Table S4. Results of permutational multivariate analysis of variance of AM fungal communities among sites and among soil and litter. This analysis used the same data that are displayed in Fig. 1c.

Supplemental Summary and Results

Using both microscopy and molecular approaches we assessed whether arbuscular mycorrhizal (AM) fungi colonize broadleaf litter within ectomycorrhizal (EM) host-dominated forests in the North American Pacific Northwest. With the molecular data, we also asked whether predominant taxa of AM fungi in the leaf litter mirrored those in the soil.

Briefly, we deployed litter bags during the wet season (winter) in two early successional forests near Bellingham, Washington, USA, which are dominated by EM Douglas fir (*Pseudotsuga menziesii*), but also include AM hosts such as bigleaf maple (*Acer macrophyllum;* hereafter 'maple') and red alder (*Alnus rubra;* Fig. 1a). We conducted two studies in different years. Study 1 was conducted with whole leaves and wide-mesh litter bags (Fig. 1a) in one forest (Forest 1), while Study 2 used leaf segments and fine-mesh litter bags (Fig. 1b) in both forests (Forests 1 and 2). Winter temperatures and precipitation in this maritime climate average 5.3 °C and 294 mm, respectively (NOAA, 2010). We used microscopy to assess densities of AM fungus-like hyphae in leaves (mm hyphae per mm^2 leaf), and molecular tools targeting the small subunit (SSU) ribosomal rRNA gene specifically to identify dominant taxa of AM fungi present inside leaf litter and underlying soil. Over the course of the two studies in different years, we used two leaf litter types: maple and *Rhapis excelsa,* a tropical Asian palm (hereafter 'palm') used in a previous study of AM fungi in leaf litter (Aristizábal, 2008). Maple litter pieces, which have reticulate venation and relatively fast rates of decomposition, were used for assessing densities of AM hyphae via light microscopy (the processed litter provided large vein-free areas and strong contrast between fungi and plant tissue facilitating identification of hyphae consistent with AM fungi). Palm litter pieces, which have parallel venation and decompose relatively slowly, provided a conservative assessment of whether AM fungi (Glomeromycota) colonized leaf litter before decomposition was visible. We extracted and amplified Glomeromycotan DNA primarily from palm samples, although we also included three composite samples of maple litter. Full methods are provided below as Supporting Information Methods.

We found microscopic and molecular evidence that AM fungi colonize leaf litter within three months of deployment in forests dominated by EM hosts (Fig. 1b, c). We observed AM fungus-like hyphae in maple leaf litter from all sites (qualitative confirmation in Study 1, and in Study 2: mean ± standard error = 4.5 ± 0.9 mm hyphae/mm^2 litter, $n = 8$) and we successfully amplified Glomeromycotan DNA from the composite maple litter samples as well as from palm leaf pieces (Supporting Information Table S2). We found a positive correlation between SOM

and AM fungal abundance in soil (SOM as loss on ignition and abundance as sequence counts; r = 0.85, t_6 = 3.9, p = 0.008). Total sequence abundance of AM fungi in soil was correlated with AM fungus-like hyphal densities in maple litter (r = 0.81, t_6 = 3.4, p = 0.015, Fig S2). Concordantly, total sequence abundance within palm litter was correlated with total sequence abundance in soil (ln[x+1]) transformed, r = 0.76, t_6 = 2.8, p = 0.030, Fig S3), but differed substantially among sites (Table S2). The correlation between sequence abundance of AM fungi in soil and leaf litter is logical given our understanding that the obligately root-associated fungi would extend between roots and leaf litter. This implies, as would be expected, that AM fungi enter leaf litter near AM host plants, but most importantly, AM fungi may enter leaf litter even in forests dominated by EM host plants.

Glomeromycotan taxa may differ in their tendency to enter leaf litter. We found the predominant AM fungi virtual taxa (VT) in soil were distinct from those in litter (based on nonmetric multidimensional ordination and permutational analysis of variance of sequence data resampled to 114 sequences/sample, Fig. 1c). Some VT found in leaf litter were not found in the underlying soil, while some VT found in soil were not found in leaf litter (Table S3); which explains why the distinction between AM fungal communities in litter and soil was significant even after taking the community variability among sites into account (Table S4). Given that AM fungi are growing from the soil into the leaf litter, any VT in leaf litter also must have been present in the soil, but likely escaped detection with our sampling effort (Fig. S4). However, VT detected only in leaf litter did not cluster phylogenetically, but instead were closely related to VT detected only in soil (Fig. S5). Thus, our results suggest that some niche differentiation may occur between the two substrates as has previously been found with EM fungi (Dickie et al, 2002), but differentiation may occur within, rather than among, phylogenetic clusters.

Full Methods S1

<u>Overview</u>

We conducted studies over two winters (2012-13 and 2015-16) in two early successional forests separated by approximately 9 km and located near Bellingham, WA, USA (Table S1). These forests are dominated by Douglas-fir (*Pseudotsuga menziesii*), but also include bigleaf maple (*Acer macrophyllum*; hereafter 'maple'), western hemlock (*Tsuga heterophylla*), red alder (*Alnus rubra)*, and western red cedar (*Thuja plicata*). Forest understory includes a mixture of

herbaceous and woody plants with a high abundance of western sword fern (*Polystichum monitum*).

Study 1: Are AM fungi colonizing litter?

In the first study we asked whether AM fungi colonize fresh leaf litter of maple (*Acer macrophyllum*). At three sites in Forest 1 (48° 44' 10" N, 122° 28' 53" W), we placed whole, recently fallen, air-dried leaves of maple (mean ± standard error; 2.0 ± 0.1 g) in ~15 x 20 cm, woven mesh, polypropylene litter bags (Fig. S1a). Five litter bags were placed along two transects at each site (2 transects x 5 positions along transects x 3 sites = 30 total litter bags). Only 19 litter bags, however, yielded useful samples; others either were lost or not enough litter material was available at harvest for analysis. Litter bags were placed on top of the mineral soil after clearing coarse litter (Fig. S1a) in late October 2012 and collected 22 weeks later. Litter was carefully removed from the bags, litter surfaces were brushed clean, and litter was air-dried and weighed to determine percent weight loss (100% x [original weight − ending weight] / original weight). Subsamples were frozen for later molecular analysis (described below) and the remaining samples were cleared, stained, and mounted on slides for qualitative assessment of colonization by AM fungi via light microscopy (described below).

Study 2: Are AM fungi colonizing leaf litter while that litter still is structurally intact?

The first study provided preliminary evidence that AM fungi were colonizing leaf litter in Forest 1. However, our samples were significantly degraded at the time of collection (mean ± standard error; 69 ± 5% weight lost based on air-dried samples), so our question of whether AM fungi would colonize fresh leaf litter, which we felt required evidence of colonization in structurally-intact litter, was not yet answered. In addition, the extent of decomposition meant that the litter was fragile, and although we were able to brush off debris, we were not able to wash litter surfaces to remove any external hyphae. Thus, we conducted a second study in which we used two leaf litter sources; maple and a plant with slowly degrading litter, *Rhapis exclesa* (hereafter 'palm'). This species is an Asian tropical palm used in previous studies of AM fungal colonization (Aristizábal, 2008). We also used finer mesh litter bags than previously to exclude roots and shredder decomposers from litter bags, and we monitored decomposition throughout

the winter by including additional 'weight-loss through time' litter bags at one site. Finally, we expanded the study into Forest 2 (48°, 39' 17" N, 122°, 27' 43" W).

The primary question of our second study was the same: 1) Are AM fungi colonizing new leaf litter? We posed additional questions, however, which were relevant in the event we found AM fungi in new leaf litter: 2) do the predominant AM fungal taxa in leaves mirror those in the soil, and 3) does AM fungal colonization of leaf litter correlate with soil organic matter of the underlying soil?

Litter bags were constructed as follows. Fresh leaves were collected in late September 2015 including middle leaflets of a palm from the University of Miami Gifford Arboretum, and senescing, but not yet fallen, leaves of maples from Cornwall Park, Bellingham, WA. Leaves were cut into 3 x 3 cm squares and dried at 60°C for 48 h. Each litter bag, made from 30 µm nylon mesh (Sefar; Buffalo, NY), included two litter samples of one litter type, either maple or palm (Fig. S1b).

In late October 2015, we placed four litter bags of each litter type along a transect at each of four new sites in Forest 1 (Sites 1-4), and at an additional four sites in Forest 2 (Sites 5-8; 2 litter types x 1 transect x 4 positions along the transect x 4 sites x 2 forests = 64 litter bags). Sites were located in groves which included mature AM hosts; either maple or red alder (*Alnus rubrus*). As in the first study, coarse litter was cleared, and litter bags were placed on the mineral soil (Fig. S1b). A thin layer of coarse litter was placed on top of the litter bags.

At the same time, we placed additional litter bags of each litter type to measure overall weight loss at all sites and to monitor weight loss through time at Site 1 (our intention was to avoid excessive litter fragmentation). These litter bags were dried at 60°C for 4 d and weighed prior to being deployed. 'Weight-loss through time' litter bags were placed in triplicate at Site 1 only (3 replicates x 1 position on the transect x 2 litter types = 6 litter bags). Overall-weight-loss litter bags were placed in triplicate along the same transect as the treatment litter bags at each site to be measured at the study's end (2 litter types x 3 positions along transects x 4 sites x 2 forests = 48 litter bags).

Maple and palm litter bags were collected after 10.5 and 16.5 weeks, respectively (compared to 22 weeks in Study 1). In the lab, litter was carefully removed from the bags, thoroughly washed with distilled water, and dried on paper towels. When sufficient material was available, subsamples were frozen for later molecular analysis (described below). Although weight loss samples to measure overall decomposition were included at all sites, only Site 1

included weight-loss samples that were collected throughout the study. These ‘weight-loss through time’ samples were used to monitor the rate of decomposition and to time sample collection. Unfortunately, decomposition rates at some sites were faster than those at Site 1, so some maple samples were fragmented and/or fragile. Among those fragmented and/or fragile samples, we prioritized samples for microscopy. Samples were mounted on slides for qualitative assessment of AM fungal colonization via light microscopy (described below).

Study 2: Soil Samples

Soil samples were collected at the time litter bags were deployed, along transects offset by ~10 cm from each litter bag transect. At each sampling location, 100 mL was collected from the center of a 10 cm × 7 cm (diameter × depth) volume of homogenized soil (4 samples x 8 sites = 32 soil samples). In the lab, subsamples were taken, pooled by site, and frozen at -20°C for later molecular analysis (see below). The remaining soil was air dried and stored at room temperature for later analysis of soil organic matter via loss on ignition (dried at 105°C for 48 hr, combusted at 500°C with a 2 hr ramp-up and 2 hr hold; reported as percent loss on ignition (100 % x [dry weight – combusted weight] / dry weight).

Studies 1 and 2: Microscopy

Maple litter was cleared and stained at room temperature using a method modified from Phillips and Hayman (1970). The first step, clearing the pigment, is a balance between achieving translucency (which allows for contrast once the sample is stained) without dissolving the sample. We monitored each sample by viewing it under a dissection microscope (daily in the first weeks, and then weekly for prolonged clearing times). Litter was cleared in 2.5-10% KOH at room temperature for a minimum of 48 h and a maximum of 30 days depending on pigmentation in the sample. As soon as samples achieved translucency, they were acidified for ~ 24 h in 3% HCl. The duration of staining differed depending on each sample and ranged from 16-20 h at room temperature in 0.05% Trypan blue in lactoglycerol. Samples were de-stained in distilled water for a minimum of 48 h prior to being mounted on slides.

Slides were made of randomly chosen inter-vein litter sections. Litter was mounted under two 1 cm^2 coverslips, and where material was available, two slides were made for each sample. When litter samples were fragile and/or fragmented, we were limited by available material and our method of choosing sections was, at best, pseudo-random. AM fungal colonization was qualitatively assessed in Study 1 slides and was quantitatively assessed in Study 2 by adapting the gridline intersect method described in Tennant (1975). Viewing the samples at 200x with a 10 x 10 cell ocular grid, we counted hyphal intersections with projected gridlines in 10 fields of view under each cover slip. The projected grid had a cell length of 50 μm and covered a sample area of 500 x 500 μm. Hyphae were considered consistent with AM fungi if they had some, but not necessarily all of the following characteristics: irregular or absent septa, non-parallel walls (often knobby in shape because of ‘unilateral angular projections’), hyphal branching at < 90°, and lack of melanization (Rillig et al, 1998). Because fungal hyphae are morphologically variable, it is possible that some hyphae we counted as ‘consistent with AM fungi’ were not AM fungi, and vice versa. Hyphae within a field of view that displayed questionable characteristics but were connected to hyphae that were consistent with those of AM fungi outside the field of view, were counted. Hyphal fragments shorter than 50 μm were ignored. A hypha terminating at, crossing, or with a curve just tangent to, a gridline was counted as one intersection, and a hypha running along a gridline was counted as two intersections. Fields of view were chosen pseudo-randomly by moving the stage arbitrary amounts in an S-shaped pattern to cover the entire subsample. If the sample occupied < 90% of the field of view, which occurred sometimes due to shape or decomposition, that field was skipped. Intersection counts were converted to hyphal length per unit leaf area. Tennant (1975) presented the following equation:

$$R = \frac{11}{14} N * g$$

where R is total length of hyphae within the grid, N is the number of intersections, and g is the grid-cell length. We converted this to density as follows:

$$D = \frac{11\,\overline{N} * g}{14\,A}$$

Where $\overline{N}$ is the mean number of intersections per field of view measured for a sample, A is the sample area covered by the grid and D is colonization density as length of hyphae per area of litter.

Studies 1 and 2: Molecular methods

We extracted DNA from both leaf litter and soil samples including two composite palm litter samples from each site (n = 2 x 8 sites = 16) and two control samples that had never been placed in the field (n = 2). We also extracted DNA from one composite soil sample from each site (n = 1 x 8 sites = 8). Maple litter was most degraded, but we were able to extract DNA from one composite maple litter sample from Study 2 and two composite maple litter samples from Study 1 (n = 3). Thus, in total, we had 31 samples available for molecular analysis, we did not find any AM fungi sequences in our two control samples, and we report the results from 29 samples in Table S2.

DNA was extracted from approximately 25 mg of lyophilized leaf tissue or approximately 250-300 mg of dried soil per sample using the MoBio PowerPlant™ and PowerSoil™ DNA isolation kits, respectively, following the manufacturer's instructions. Samples were prepared for Illumina sequencing using a two-step PCR protocol to first amplify our target region and then attach unique sample identifiers. For this study we used the AM fungi specific primers WANDA (Dumbrell et al, 2011) and AML2 (Lee et al, 2008) to target the small subunit (SSU) rRNA. Detailed descriptions of the PCR protocols used can be found in McTee et al. (2017) and Lekberg et al. (2018). Briefly, all PCR1 reactions were carried out in 12.5 μL reaction volumes containing 1 μL of DNA extract as template, and 20.0 pmol of each primer in 1X GoTaq® Green Master Mix [(Green GoTaq® Reaction Buffer, 200 μM dATP, 200 μM dGTP, 200μM dCTP, 200μM dTTP and 1.5mM $MgCl_2$) Promega, USA]. Each reaction was performed singly or in duplicate to ensure adequate amplification in a Techne TC-4000 thermocycler (Bibby Scientific, Burlington, USA). Amplicons generated during PCR1 were diluted 1:10 for use as template in PCR2. PCR2 then was carried out following Bullington et al. (2018). PCR2 amplicons were pooled based on band intensity and purified using AMPure XP beads (Beckman Coulter Genomics, USA) prior to sequencing. Sequencing was done at the Institute for Bioinformatics and Evolutionary Studies (IBEST) genomics resources core at the University of Idaho (http://www.ibest.uidaho.edu/; Moscow, ID, USA). Amplicon libraries were sequenced using ¼ of a 2 x 300 paired-end (PE) run on an Illumina MiSeq sequencing platform (Illumina Inc., San Diego, CA, USA).

Studies 1 and 2: Bioinformatics

Bioinformatics analyses were performed using "Quantitative insights into microbial ecology 2", (QIIME2 version 2018.2; https://qiime2.org/; Boylen et al, 2018). Sequence reads were first demultiplexed using the q2-demux plugin (https://github.com/qiime2/q2-demux). Only forward reads were used for the SSU region, as the overlap between forward and reverse reads is too short to merge the two without significant sequence loss. Forward reads were trimmed to 210 bp, which covers the informative region of our 18S target (Lee et al, 2008), quality filtered, and de-replicated using the q2-dada2 plugin (Callahan et al, 2016). The q2-dada2 plugin uses nucleotide quality scores to produce sequence variants (SVs), or sequence clusters with 100% similarity representing the estimated true biological variation within each sample. Although sequences are clustered at 100% similarity as opposed to the traditional 97% similarity, DADA2 produces fewer spurious sequences, fewer clusters, and results in a more accurate representation of the true biological variation present (Callahan et al, 2016). This original quality filtering resulted in 79,673 sequences. Because this region and primers can amplify non-target DNA (Lekberg & Bullington, personal observation), we used several approaches to ensure that we only retained sequences of AM fungi for downstream analyses. We removed all SVs that did not match virtual taxa in MaarjAM, a reference database for AM fungi (Öpik et al, 2010), with at least 80% similarity and coverage (https://github.com/qiime2/q2-quality-control). In previous analyses we have found 80% to be a conservative cutoff for removing non-target DNA within this region. This resulted in the removal of 286 non-target SVs (49,409 sequences) while 101 SVs (30,264 sequences) were retained. The QIIME2 q2-feature-classifier (https://github.com/qiime2/q2-feature-classifier), a naive Bayes machine-learning classifier which has been shown to meet or exceed classification accuracy of existing methods (Bokulich et al, 2017), then was used to assign taxonomy for those remaining reads. We set a confidence threshold of 0.94 and used a previously altered version of the MaarjAM database that included many non-target SSU reads to limit over classification of sequences to AM fungi. This allowed us to identify and remove many additional non-target sequences amplified by the WANDA and AML2 primers. All remaining SVs were aligned with all virtual taxa sequences from MaarjAM with the QIIME2 alignment plugin (MAFFT, Katoh & Standley, 2013), removing non-informative nucleotide positions without at least one character present in at least 40% of sequences. We produced a midpoint-rooted phylogenetic tree from this alignment using the QIIME2 phylogeny plugin (FastTree 2, Price et al, 2010) to verify identification as AM fungi. These steps additionally were performed

on all previously removed 'non-target' sequences to confirm that they were not AM fungi. Our quality control efforts identified a total of 69 AM fungus SVs, represented by 18,633 sequences in both soil and leaf tissue (Table S2), or 23% of the original 79,673 original quality filtered sequences. SVs were further collapsed based on MaarjAM virtual taxa assignments. Species accumulation curves were produced using the function "specaccum" in the 'vegan' R package and data were rarefied at 114 sequences per sample for even sampling depth (Fig. S4). A final neighbor-joining tree was produced from the rarefied data using the function "nj" in the R package 'ape' (Paradis et al, 2004) to illustrate the phylogenetic differences between sequences recovered from litter and soil (Fig. S5). All sequences have been submitted to GenBank and assigned accession numbers MH453967-MH454026.

Studies 1 and 2: Statistical Analyses

All statistical analyses were completed using the statistical software R (version 3.4.4; R Core Team, 2018) in the RStudio environment (version 1.1.442, RStudio Team, 2016). Correlations and t-tests were completed using the "cor.test" and "t.test" functions from the base stats package after confirming data met assumptions of normality and homoscedasticity. We used nonmetric multidimensional scaling (NMDS) to evaluate similarities among predominant AM fungal taxa in leaf litter and soil. Only samples with sufficient sequences (>114 sequences/sample) were included in the ordination: these included soil samples from all sites of Study 2, palm litter samples from Sites 5, 6, and 8 in Forest 2 of Study 2, and three pooled maple litter samples (two from Forest 1 in Study 1 and one from Forests 1 and 2 in Study 2). These data were reduced to two dimensions via NMDS with the "metaMDS" function and a euclidean dissimilarity matrix in the vegan package (Oksanen et al, 2018). Data reduction produced very low stress (0.09), and the nonmetric r^2 between the ordination distance and observed dissimilarity was 0.99. To determine if AM fungal communities differed between soil and litter while accounting for differences among sites, we analyzed our dissimilarity matrix as follows. We first checked for multivariate homogeneity of dispersion among soil and litter communities and among site communities using "betadisper" and "anova." We found no evidence against homogeneity ($F_{1,12}$=1.63, p=0.23; $F_{8,5}$=1.40, p=0.37; respectively). We then proceeded with permutational multivariate analysis of variance using the "adonis2" function including first 'site' and then 'soil or litter' as sequential factors.

References

Aristizábal C. (2008). Arbuscular mycorrhizal fungi enhance the acquisition of mineral nutrients from leaf litter by *Morella cerifera*. PhD thesis. Miami, Florida: University of Miami.

Bokulich N, Kaehler B, Rideout J, Dillon M, Bolyen E, Knight R, et al. (2017). Optimizing taxonomic classification of marker gene sequences. *PeerJ Preprints* doi:5:e3208v1.

Bolyen E, Rideout JR, Dillon MR, Bokulich, NA, Abnet C, Al-Ghalith, GA, et al. (2018) QIIME 2: Reproducible, interactive, scalable, and extensible microbiome data science. *PeerJ Preprints* doi: 10.7287/peerj.preprints.27295v2.

Bullington LS, Lekberg Y, Sniezko R, Larkin B. (2018). The influence of genetics, defensive chemistry and the fungal microbiome on disease outcome in whitebark pine trees. *Molecular Plant Pathology* doi: 10.1111/mpp.12663

Callahan BJ, McMurdie PJ, Rosen MJ, Han AW, Johnson AJA, Holmes SP. (2016). DADA2: High-resolution sample inference from Illumina amplicon data. *Nature Methods* 13: 581–583.

Dickie IA, Xu B, Koide RT. (2002). Vertical niche differentiation of ectomycorrhizal hyphae in soil as shown by T-RFLP analysis. *New Phytologist* 156: 527-535.

Dumbrell AJ, Ashton PD, Aziz N, Feng G, Nelson M, Dytham C, et al. (2011). Distinct seasonal assemblages of arbuscular mycorrhizal fungi revealed by massively parallel pyrosequencing. *New Phytologist* 190: 794–804.

Katoh K, Standley DM. (2013). Mafft multiple sequence alignment software version 7: improvements in performance and usability. *Molecular Biology and Evolution* 30: 772-780. doi:10.1093./molbev/mst010.

Lee J, Lee S, Young JPW. (2008). Improved PCR primers for the detection and identification of arbuscular mycorrhizal fungi. *FEMS Microbiology Ecology* 65: 339–349.

Lekberg Y, Vasar M, Bullington L, Sepp S-K, Antunes P, Bunn R, et al. (2018). More bang for the buck? Can arbuscular mycorrhizal fungal communities be characterized adequately alongside other fungi using general fungal primers? *New Phytologist* 220: 971-976. doi:10.1111/nph.15035

McTee MR, Lekberg Y, Bullington L, Rummel A, Mummey D, Ramsey PW, Hinman N. (2017). Restoring ecological properties of acidic soils contaminated with elemental sulfur. *Science of the Total Environment*. 587-588: 449-456. doi:10.1016/j.scitotenv.2017.02.110.

NOAA. (2010). 1981-2010 U.S. Climate Normals. [WWW document] URL https://www.ncdc.noaa.gov/data-access/land-based-station-data/land-based-datasets/climate-normals/1981-2010-normals-data [accessed 1 March 2018].

Oksanen, AJ, Blanchet FG, Friendly M, Kindt R, Legendre P, McGlinn D, et al. (2018). vegan: Community Ecology Package. R package version 2.5-1. https://CRAN.R-project.org/package=vegan

Öpik M, Vanatoa A, Vanatoa E, Moora M, Davison J, Kalwij JM, et al. (2010). The online database MaarjAM reveals global and ecosystemic distribution patterns in arbuscular mycorrhizal fungi (Glomeromycota). *New Phytologist* 188: 223–241.

Paradis E, Claude J, Strimmer K. (2004). APE: Analyses of Phylogenetics and Evolution in R language. *Bioinformatics* 20: 289–290.

Phillips JM, Hayman DS. (1970). Improved procedures for clearing roots and staining parasitic and vesicular-arbuscular mycorrhizal fungi for rapid assessment of infection. *Transactions of the British Mycological Society* 55: 158–161.

Price MN, Dehal PS, Arkin AP. (2010). FastTree 2 – Approximately maximum-likelihood trees for large alignments. PLOS One 5: e9490. doi:10.1371/journal.pone.0009490.

R Core Team. (2018). R: A language and environment for statistical computing. R Foundation for Statistical Computing, Vienna, Austria. URL https://www.R-project.org/.

Rillig MC, Allen MF, Klironomos JN, Field CB. (1998). Arbuscular mycorrhizal percent root infection and infection intensity of *Bromus hordeaceus* grown in elevated atmospheric CO_2. *Mycologia* 90: 199–205.

RStudio Team. (2016). RStudio: Integrated Development for R. RStudio, Inc., Boston, MA URL http://www.rstudio.com/.

Tennant D. (1975). A Test of a Modified Line Intersect Method of Estimating Root Length. *Journal of Ecology* 63: 995–1001.

Supplemental Figures

Figure S1. a) Woven, polypropylene mesh litter bags (~15 x 20 cm) containing entire bigleaf maple (*Acer macrophyllyum*) leaves were placed beneath loose litter for our first study, Study 1, in which all sites were located in Forest 1. b) Litter bag placement was similar in our second study, Study 2, but bags were smaller (~3 x 3 cm) and made of 30 μm nylon mesh. This study used sites in Forests 1 and 2.

(a)

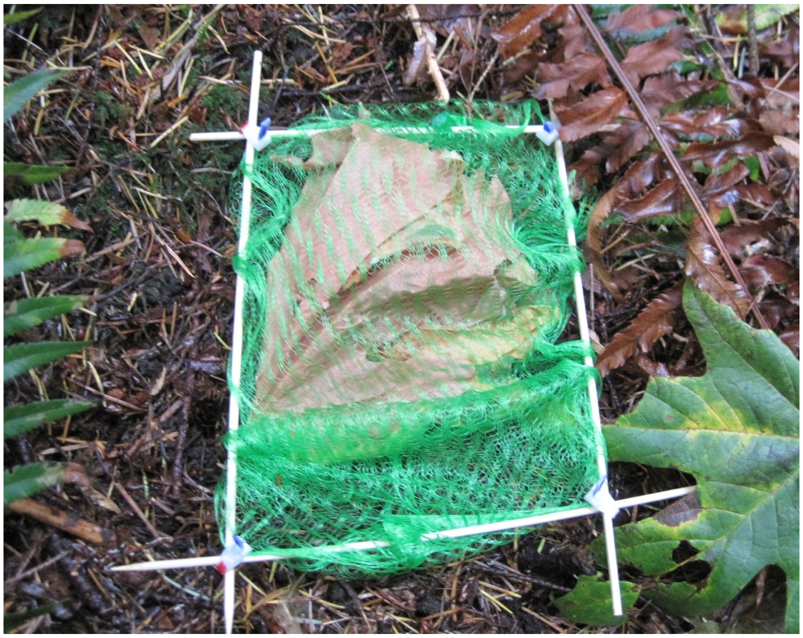

(b)

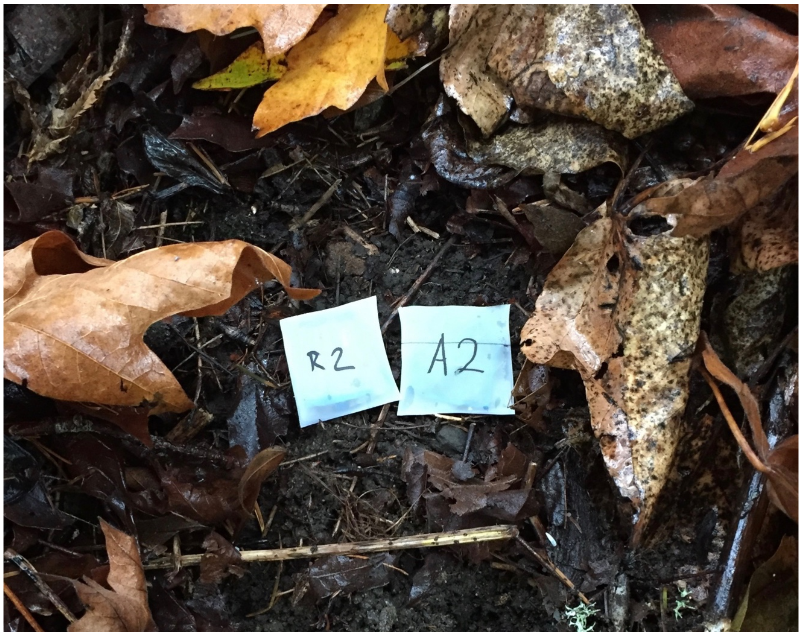

Figure S2. AM fungal sequence abundance in soil samples plotted against AM fungal-like hyphal densities determined microscopically in overlying bigleaf maple (*Acer macrophylum*) leaf litter samples from our second study, Study 2. Each point on the graph represents paired measurements from one of our eight study sites and the correlation coefficient was estimated using Pearson's method. Raw sequence data are presented in Table S2, but hyphal densities are reported here only. This plot suggests that AM fungus colonization of leaf litter is driven in part by densities of AM fungi in the soil. Yet, the plot should be interpreted with caution as hyphal morphology is variable, and distinguishing AM fungal hyphae from other fungal hyphae in the soil is difficult; therefore, measured hyphal densities might include some non-AM fungi hyphae or conversely, exclude some AM fungi.

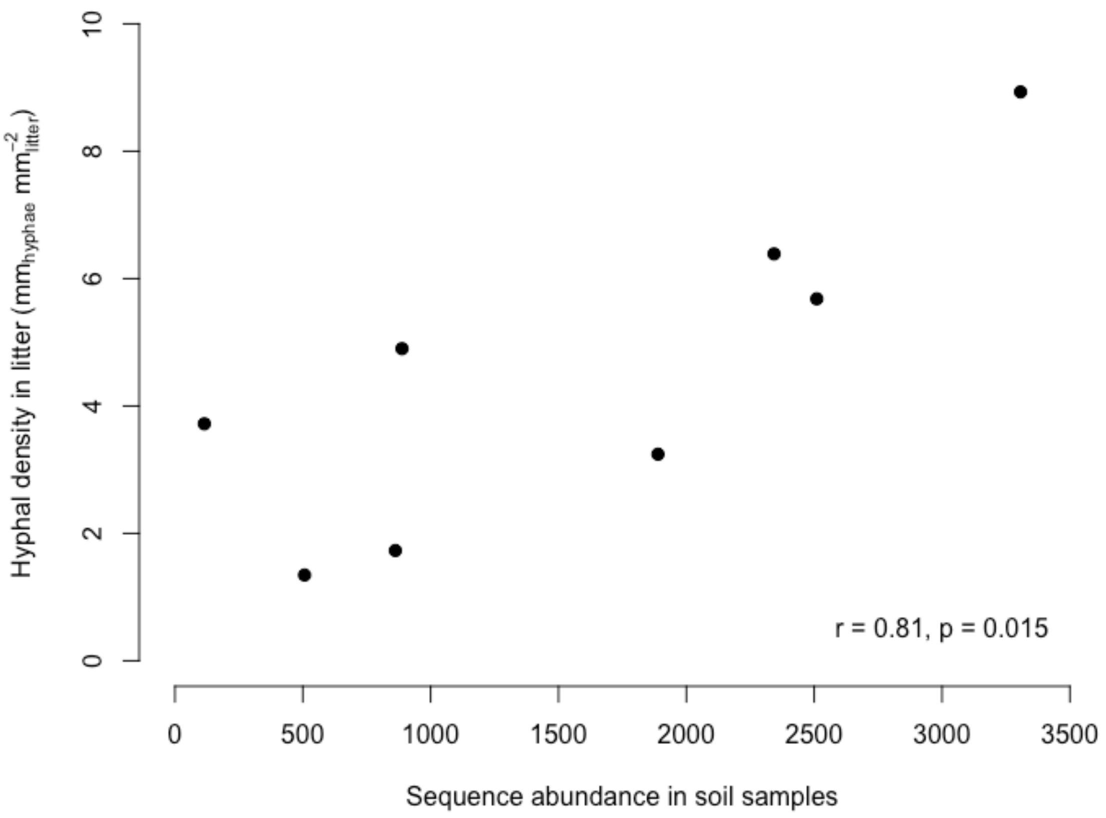

Figure S3. AM fungal sequence abundance in soil samples plotted against AM fungal sequence abundances in overlying palm (*Rhapis excelsa*) leaf litter samples from our second study, Study 2. Each point on the graph represents paired measurements from one of our eight study sites. Raw sequence data are presented in Table S2. Note that values on the vertical axis were ln(x+1) transformed to improve normality and the correlation coefficient was estimated using Pearson's method. This plot suggests that AM fungus colonization of leaf litter is driven in part by densities of AM fungi in the soil, although the relationship is nonlinear.

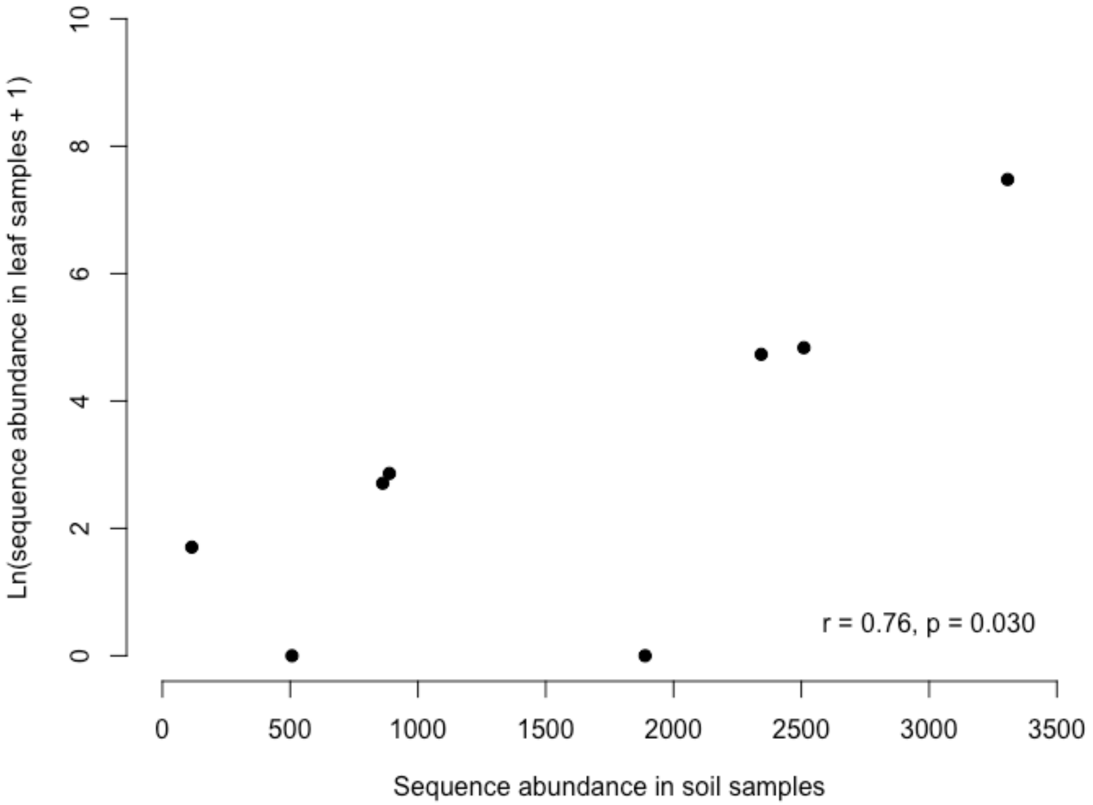

Figure S4. The three species accumulation curves represent all samples combined (combined; diamonds), leaf litter samples only (litter; triangles), and soil samples only (soil; squares). These curves, which are not asymptotic, suggest additional samples would have revealed more species of arbuscular mycorrhizal fungi in both source materials. Curves are based on resampled data which was rarefied to 114 sequences per sample for even sampling depth. The full dataset (pre-rarefication) is available in Table S2. Shaded polygons represent +/- one standard deviation from the line.

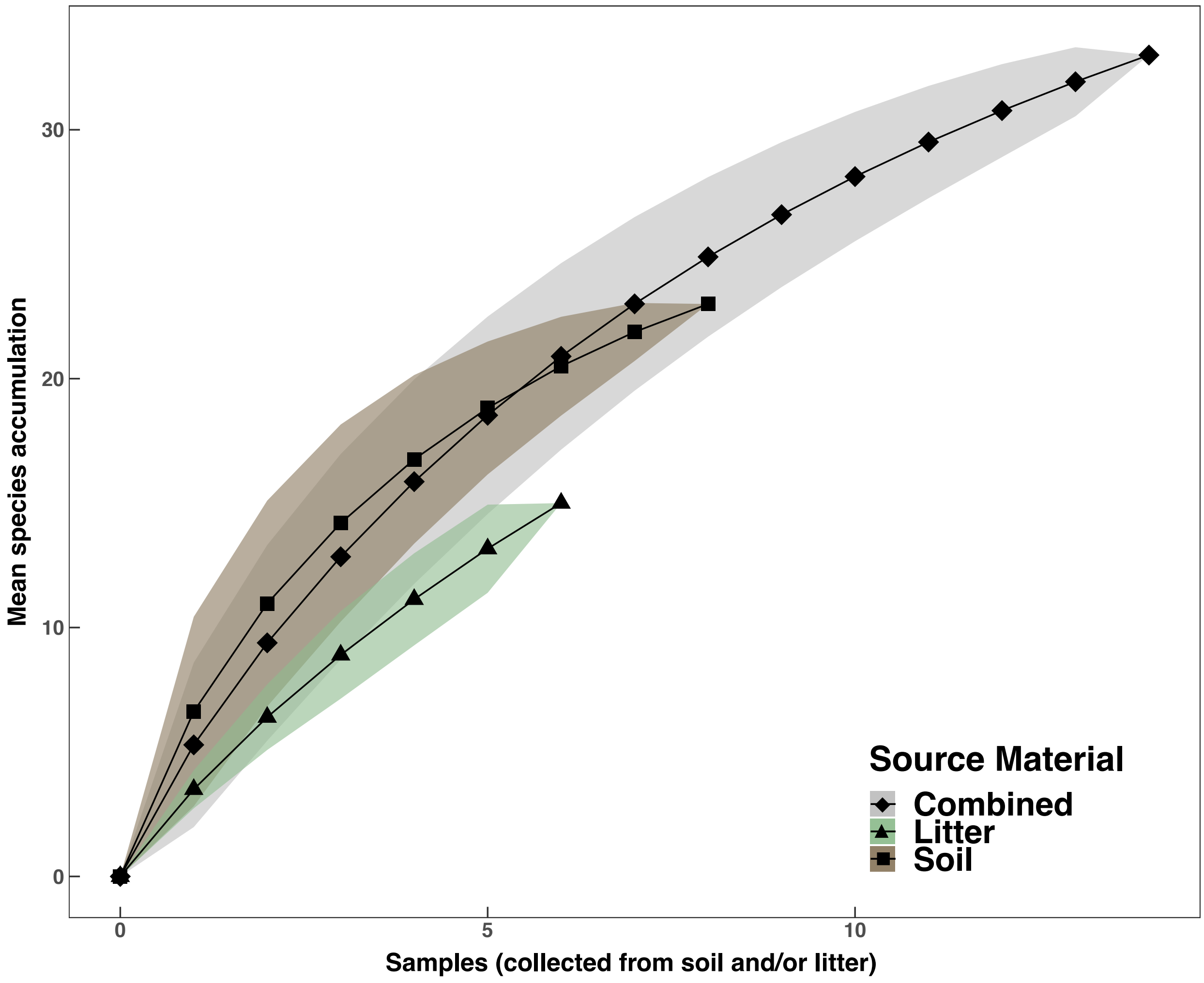

Figure S5. Phylogenetic differences among sequences recovered from litter and soil are shown in this neighbor-joining tree. Shapes denote which sequences were recovered from litter only (triangles), soil only (squares), or litter and soil (circles), while colors denote families of arbuscular mycorrhizal fungi. This tree is based on resampled data which were rarefied to 114 sequences per sample for an even sampling depth. The full dataset (pre-rarefication) is available in Table S2.

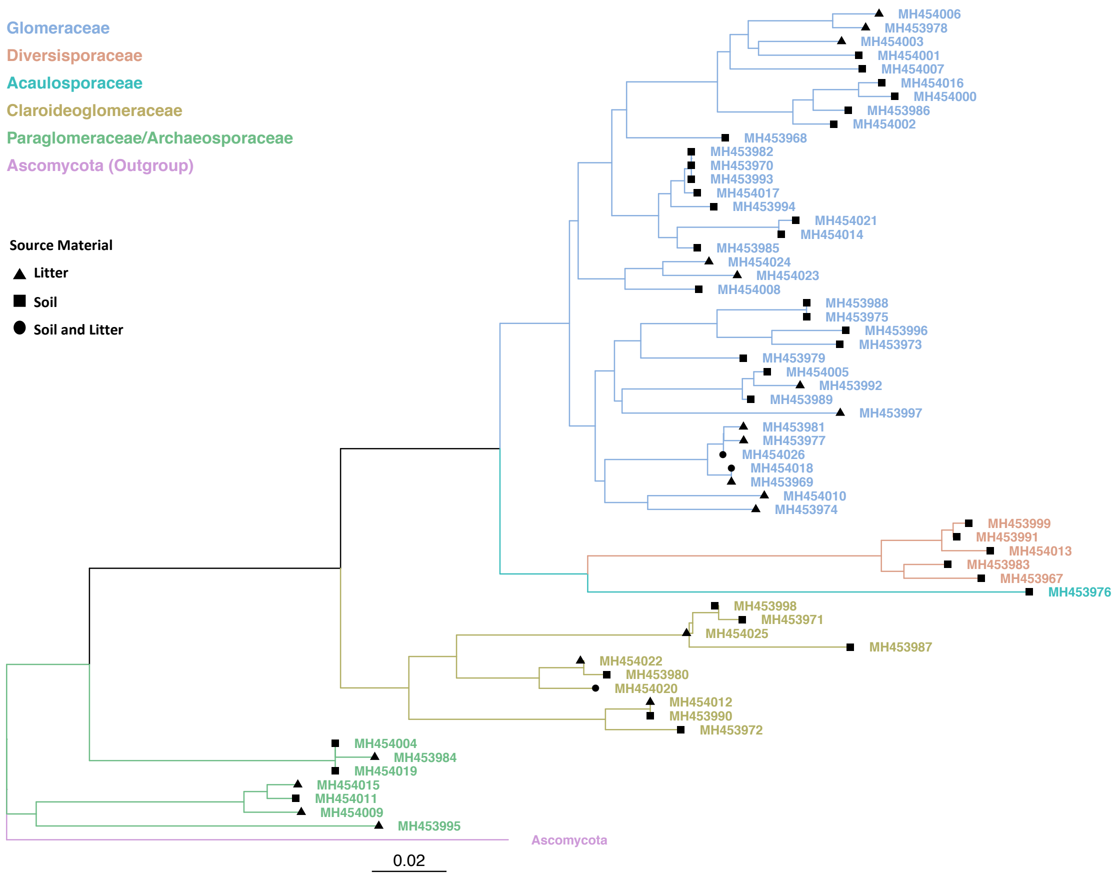

Supplemental Tables located in separate files.
