# Supplementary material for: Revisiting the ‘direct mineral cycling’ hypothesis: arbuscular mycorrhizal fungi colonize leaf litter, but why?
Source: ISME J. 2019 Mar 25;13(8):1891–8. doi: 10.1038/s41396-019-0403-2 (PMC6775977; doi:10.1038/s41396-019-0403-2)
Supplement: Supplementary file 2 — Tables S1, S3, and S4 [file 41396_2019_403_MOESM2_ESM.pdf]

Table S1. Study sites were located in two mixed maritime forests in the early stages of succession. These two forests, which we called Forest 1 and Forest 2, are approximately 9 km apart and are located near Bellingham, Washington, USA. Altitude and soil organic matter content of the studied forests are presented as means ± standard errors. Study sites 1 through 4 were located in Forest 1, while 5 through 8 were located in Forest 2.

Location ID	n	Altitude (m)	Organic matter (%)	Study Sites
Forest 1	4	174	14.4 ± 3.8	1- 4, composite
Forest 2	4	471	24.5 ± 5.0	5 - 8

Table S3. Arbsucular mycorrhizal fungal taxa found in litter, soil, or both litter and soil after resampling to 114 sequences/sample.

Where found	Virtual taxa	Genus
Only in leaf litter		
	VTX00067	*Glomus*
	VTX00108	*Glomus*
	VTX00113	*Glomus*
	VTX00115	*Glomus*
	VTX00143	*Glomus*
	VTX00165	*Glomus*
	VTX00197	*Glomus*
	VTX00159	*Glomus*
	VTX00239	*Paraglomus*
In both leaf litter and soil		
	VTX00004	*Archaeospora*
	VTX00057	*Claroideoglomus*
	VTX00225	*Claroideoglomus*
	VTX00357	*Claroideoglomus*
	VTX00160	*Glomus*
	VTX00281	*Paraglomus*
	VTX00308	*Paraglomus*
Only in soil		
	VTX00030	*Acaulospora*
	VTX00056	*Claroideoglomus*
	VTX00193	*Claroideoglomus*
	VTX00340	*Claroideoglomus*
	VTX00061	*Diversispora*
	VTX00062	*Diversispora*
	VTX00072	*Glomus*
	VTX00074	*Glomus*
	VTX00084	*Glomus*
	VTX00088	*Glomus*
	VTX00122	*Glomus*
	VTX00129	*Glomus*
	VTX00153	*Glomus*
	VTX00199	*Glomus*
	VTX00214	*Glomus*
	VTX00216	*Glomus*
	VTX00219	*Glomus*

Table S4. Results of a permutational multivariate analysis of variance of AM fungal communities considering two factors, site and source of mycorrhizal community (*i.e.* soil or litter). This analysis used the same data that are displayed in Fig. 1c. Note that the factor 'site' included a separate category for composite samples.

Source	df	SS	R^2	F	p
site	8	39418	0.698	2.86	0.006
soil/litter	1	10127	0.179	5.88	0.004
residual	4	6891	0.122		
Total	13	56436			
